# Retrospective analysis of pneumothorax after repair of esophageal atresia/tracheoesophageal fistula

**DOI:** 10.1186/s12887-021-02948-x

**Published:** 2021-12-03

**Authors:** Jiawei Zhao, Shen Yang, Siqi Li, Peize Wang, Yanan Zhang, Yong Zhao, Kaiyun Hua, Yichao Gu, Junmin Liao, Shuangshuang Li, Yongwei Chen, Jinshi Huang

**Affiliations:** Department of Neonatal Surgery, Beijing Children’s Hospital, Capital Medical University, National Center for Children’s Health, 100045 Beijing, China

**Keywords:** Esophageal atresia, Pneumothorax, Anastomotic leakage, Mechanical ventilation

## Abstract

**Background:**

To analyze the possible causes, treatment and outcomes of postoperative pneumothorax in patients with Gross type C esophageal atresia/tracheoesophageal fistula (EA/TEF).

**Methods:**

Medical records of patients with Gross type C EA/TEF who were diagnosed and treated in Beijing Children’s Hospital from January 2007 to January 2020 were retrospectively collected. They were divided into 2 groups according to whether postoperative pneumothorax occurred. Univariate and multivariate logistic regression analysis were performed to identify risk factors for pneumothorax.

**Results:**

A total of 188 patients were included, including 85 (45 %) in the pneumothorax group and 103 (55 %) in the non-pneumothorax group. Multivariate logistic regression analysis showed that postoperative anastomotic leakage [*P* < 0.001, OR 3.516 (1.859, 6.648)] and mechanical ventilation [*P* = 0.012, OR 2.399 (1.210, 4.758)] were independent risk factors for pneumothorax after EA/TEF repair. Further analysis of main parameters of mechanical ventilation after surgery showed that none of them were clearly related to the occurrence of pneumothorax. Among the 85 patients with pneumothorax, 33 gave up after surgery and 52 received further treatment [conservative observation (n = 20), pleural puncture (n = 11), pleural closed drainage (n = 9), both pleural puncture and closed drainage (n = 12)]. All of the 52 patients were cured of pneumothorax at discharge.

**Conclusions:**

Anastomotic leakage and postoperative mechanical ventilation were risk factors for pneumothorax after repair of Gross type C EA/TEF, but the main parameters of mechanical ventilation had no clear correlation with pneumothorax. After symptomatic treatment, the prognosis of pneumothorax was good.

**Supplementary Information:**

The online version contains supplementary material available at 10.1186/s12887-021-02948-x.

## Introduction

Esophageal atresia/tracheoesophageal fistula (EA/TEF) is a relatively common neonatal gastrointestinal tract malformation with an incidence of 1/4000-1/2500 [1], once diagnosed, surgical treatment is required. Previous studies have reported that common complications after repair of EA/TEF include anastomotic leakage (2.5–28.6 %) [2–4], anastomotic stricture (8.7–60 %) [5–8], recurrent tracheoesophageal fistula (8.9–19 %) [9–12], etc. However, pneumothorax has not attracted enough attention as a common complication after EA/TEF repair with an incidence rate of 23.8–37.9 % [13]. Thus, this study aims to summarize the possible causes, diagnosis, treatment strategies, and outcomes of pneumothorax after EA/TEF repair by reviewing the clinical data of children with Gross type C EA/TEF.

## Materials and methods

### Patients

The medical records of patients with Gross type C EA/TEF diagnosed and treated in Beijing Children’s Hospital from January 2007 to January 2020 were retrospectively collected, and all of them underwent one-stage surgical repair (the patients with long-gap type C EA/TEF were excluded). Relevant information, including age, sex, birth weight, combined deformities, perioperative conditions, mechanical ventilation parameters, postoperative complications were extracted from electronic medical records and follow-up was performed. In this study, pneumothorax was defined as clinically significant pneumothorax within 2 weeks after the surgery, which was diagnosed by clinicians in combination with the patients’ symptoms (rapid breathing, distress, decreased blood oxygen saturation), signs (absence or decrease of respiratory sounds on the affected side, percussion drum sound) and chest radiographs. Patients were divided into 2 groups according to whether pneumothorax occurred, and the differences in clinical characteristics and perioperative conditions between the 2 groups were compared. This study was approved by the Medical Ethics Committee of Beijing Children’s Hospital (2019-K-333) and waived patients from the requirement of informed consent.

### Statistical methods

SPSS 23.0 statistical software was used for analysis. Continuous variables were presented as the mean with standard deviation or median and interquartile range if the normality hypothesis test rejected the null hypothesis of normal distribution. Categorical variables were reported as counts and percentages. Pearson’s χ2 test, Fisher’s exact test, two independent samples t-tests and the non-parametric Mann-Whitney U test were used to compare characteristics between the groups. *P* < 0.05 indicated a statistically significant difference.

## Results

### Patient characteristics

A total of 188 patients with Gross type C EA/TEF were included in this study, including 130 males (69 %) and 58 females (31 %). The median age at operation was 4 (3, 7) days, and the mean birth weight was (2.92 ± 0.50) kg. One hundred and fifty-four patients had other malformations [cardiovascular system (n = 150), motor system (n = 21), digestive system (n = 14), genitourinary system (n = 12), respiratory system (n = 10)]. Fifty-five patients (29 %) and 127 patients (68 %) underwent thoracoscopic and open surgery, with 98 patients (52 %) and 90 patients (48 %) underwent extrapleural and intrapleural approach respectively (as shown in Supplementary Table 1). Six patients (3 %) were converted to open surgery due to high anastomotic tension or decreased oxygen saturation. The main postoperative complications included pneumothorax (n = 85, 45 %), anastomotic leakage (n = 74, 39 %), anastomotic stricture (n = 66, 35 %), and recurrent tracheoesophageal fistula (n = 13, 7 %). After a median follow-up time of 68 (22, 117) months, 122 patients survived, 11 patients died (6 died in hospital after giving up treatment, 5 died of respiratory failure and infection), and 55 patients were lost to follow-up (including 37 patients who gave up treatment after surgery).

### Risk factors of pneumothorax after EA/TEF repair

According to the occurrence of postoperative pneumothorax, the patients in this study were retrospectively divided into pneumothorax group (85, 45 %) and non-pneumothorax group (103, 55 %), and the clinical characteristics and perioperative conditions of the 2 groups were compared. As shown in Table 1, we found that the differences in postoperative anastomotic leakage (*P* < 0.001) and mechanical ventilation (*P* = 0.025) were statistically significant, while there were no significant differences in other clinical features between the 2 groups (*P* > 0.05). Multivariate logistic regression analysis showed that postoperative anastomotic leakage [*P* < 0.001, OR 3.516 (1.859, 6.648)] and mechanical ventilation [*P* = 0.012, OR 2.399 (1.210, 4.758)] were independent risk factors for pneumothorax after EA/TEF repair (Table 2).

**Table 1:** Clinical comparison between pneumothorax and non-pneumothorax groups

Variables		Pneumothorax group (n = 85)	Non-pneumothorax group (n = 103)	*P*
Operation age (median, days)		4 (3, 7)	5 (3, 7)	0.251
Birth weight (mean, kg)		2.92 ± 0.48	2.92 ± 0.51	0.970
Sex (n, %)	Male	55 (64.7)	75 (72.8)	0.231
	Female	30 (35.3)	28 (27.2)	
Multiple malformations (n, %)	Yes	7 (8.4)	8 (7.8)	0.868
	No	76 (91.6)	95 (92.2)	
Distance between proximal and distal pouches (mean, cm)		1.79 ± 1.09	1.58 ± 0.91	0.175
Operation method (n, %)	Thoracoscopy	23 (28.4)	32 (31.7)	0.631
	Open surgery	58 (71.6)	69 (68.3)	
Operation approach (n, %)	Extrapleural	47 (55.3)	51 (49.5)	0.430
	Intrapleural	38 (44.7)	52 (50.5)	
Operation time (median, minutes)		125 (101, 165)	120 (105, 160)	0.943
Anastomotic leakage (n, %)	Yes	45 (54.9)	29 (28.2)	< 0.001^a^
	No	37 (45.1)	74 (71.8)	
Postoperative mechanical ventilation (n, %)	Yes	65 (76.5)	63 (61.2)	0.025^a^
	No	20 (23.5)	40 (38.8)	
Duration of postoperative mechanical ventilation (mean, hours)		134 (48, 179)	135 (50, 190)	0.676

^a^The difference was statistically significant

**Table 2:** Multivariate logistic regression analysis of risk factors for pneumothorax

Variables	B	Standard Error	Wald	*P*	OR (95 % CI)
Anastomotic leakage	1.257	0.325	14.960	< 0.001^a^	3.516 (1.859, 6.648)
Postoperative mechanical ventilation	0.875	0.349	6.273	0.012^a^	2.399 (1.210, 4.758)

^a^The difference was statistically significant

### The possible relationship between mechanical ventilation and pneumothorax

In order to explore the relationship between mechanical ventilation and pneumothorax, 78 patients who entered intensive care unit (ICU) after surgery and received mechanical ventilation who did not give up treatment were enrolled into the further analysis of ventilator parameters when pneumothorax appeared. These patients were divided into 2 groups as combined with anastomotic leakage (n = 18) and without anastomotic leakage (n = 60). As shown in Table 3, results showed that among the patients without anastomotic leakage, inspiration rate (*P* = 0.005) and peak inspiratory pressure (PIP, *P* = 0.028) were higher in the non-pneumothorax group. However there were no significant differences in positive end-expiratory pressure (PEEP, *P* = 0.858) and fraction of inspiration O_2_ (FiO_2_, *P* = 0.224) between the 2 groups. In patients with anastomotic leakage, there were no statistically significant differences in ventilator parameters between the 2 groups (all *P* > 0.05). Therefore, these results did not support the correlation between mechanical ventilation parameters and occurrence of pneumothorax after EA/TEF repair.

**Table 3:** Comparison of mechanical ventilation parameters between pneumothorax and non-pneumothorax groups

Ventilator parameters	Patients without anastomotic fistula		*P*	Patients with anastomotic fistula		*P*
	Pneumothorax (n = 22)	Non-pneumothorax (n = 38)		Pneumothorax (n = 10)	Non-pneumothorax (n = 8)	
Inspiration rate (median, times/minutes)	40 (35, 40)	45 (40, 48)	0.005^a^	38 (25, 50)	40 (40, 50)	0.236
Peak inspiratory pressure (mean, cmH_2_O)	16.05 ± 2.01	17.57 ± 2.68	0.028^a^	14.00 ± 2.60	16.88 ± 2.48	0.161
Positive end-expiratory pressure (median, cmH_2_O)	5.50 (4.25, 6.00)	5.20 (4.00, 6.25)	0.858	4.00 (3.22, 5.00)	5.30 (4.23, 6.28)	0.090
Fraction of inspiration O_2_ (median, %)	30 (25, 35)	30 (30, 40)	0.224	30 (21, 50)	33 (28, 40)	0.788

^a^The difference was statistically significant

### The treatment strategies and outcomes of patients with pneumothorax

Among the 85 patients with pneumothorax in this study, 33 gave up treatment after surgery (reasons included postoperative complications, economic embarrassment, multiple malformations, etc.), 52 patients received further treatment [conservative observation (n = 20), pleural puncture (n = 11), pleural closed drainage (n = 9), both pleural puncture and closed drainage (n = 12)]. All of the 52 patients were cured of pneumothorax at discharge, 45 patients survived during follow-up, 1 patient died out of hospital due to respiratory failure, and 6 patients were lost to follow-up.

## Discussion

Pneumothorax is a common complication after EA/TEF repair, the incidence of pneumothorax in this cohort is 45 %. Severe pneumothorax can affect the respiratory and circulatory system, even endanger life. The treatment strategies of pneumothorax include conservative observation, thorax puncture, and thoracic drainage, and most patients can often be cured. However, the pathogenesis of pneumothorax after EA/TEF repair has rarely been discussed in previous reports. In this study, we reviewed the clinical data of 188 patients with Gross type C EA/TEF and found that anastomotic leakage and mechanical ventilation were independent risk factors for pneumothorax. However, mechanical ventilator parameters could not be considered as being related to pneumothorax.

Anastomotic leakage, one of the complications with a high incidence after EA/TEF repair [14], is closely related to pneumothorax, and our data also supports this view. The clinical manifestations of anastomotic leakage are sometimes similar to pneumothorax, such as dyspnea and decreased blood oxygen, which can be identified by chest radiograph or esophagography. However, the coexistence of these two complications is not conducive to the prognosis of these patients [13]. Therefore, close attention should be paid to whether patients with anastomotic leakage have pneumothorax at the same time; and patients with pneumothorax should also be alert to whether anastomotic leakage has occurred at the same time. Postoperative thoracic drainage has become a routine operation, but some scholars believe that it can’t reduce the incidence of postoperative respiratory complications and mortality [13], nor can it prevent the occurrence of pneumothorax and pleural effusion [15], even in 10 % of patients had no clinical significance in postoperative indwelling thoracic drainage [16]. In this study, thoracic drainage tubes were routinely placed in all patients after intraexpleural surgery, which we believe was beneficial for some patients to observe the characteristics of thoracic drainage fluid. However, the issue of prophylactic thoracic drainage after EA/TEF repair still needs to be explored in prospective studies.

Previous studies have shown that there is a certain relationship between mechanical ventilation and pneumothorax [17, 18], but the causes of pneumothorax have not been analyzed in detail, and the sample size is very small. In this study, the results suggested that mechanical ventilation was one of the possible risk factors for pneumothorax, and we further compared and analyzed the specific parameters of mechanical ventilation. However, the results did not support the correlation between mechanical ventilation parameters and occurrence of pneumothorax. In addition, there was no significant relationship between mechanical ventilation and anastomotic leakage, as shown in Supplementary Table 2. Regarding the inconsistencies from the above results, we consider that it may be associated with the following factors. First, the patients of earlier years (before 2014) did not routinely receive mechanical ventilation after EA/TEF repair; only some patients with certain complications, respiratory failure, and high anastomosis tension would have received mechanical ventilation. Therefore, bias might exist in the comparison results of these patients. In addition, postoperative mechanical ventilation is a continuous treatment process, and the parameters of mechanical ventilation would have also been constantly adjusted according to the patients’ conditions. In this study, parameters were selected when pneumothorax appeared, but the parameters at other time points might differ between the pneumothorax and the non-pneumothorax groups. Greenough [19] believed that neonates should be subjected to simultaneous mechanical ventilation at a lower PIP to reduce lung trauma, air leakage, bronchopulmonary dysplasia and other problems. Horn [20] found that high PEEP would lead to reduced blood perfusion in the diaphragmatic muscle, thus affecting respiration in animal experiments. In summary, the relationship between mechanical ventilation and pneumothorax still needs further research.

Some scholars [17] believe that the application of continuous positive airway pressure (CPAP) during the withdrawal period or after the removal of endotracheal intubation might be related to the production of pneumothorax, but only 2 cases were mentioned in their study, and the values of PEEP and FiO_2_ were not provided. Diez [18] reported 3 patients with pneumothorax after tracheal intubation and CPAP, and the PEEP values were 5, 6 and 6 cmH_2_O respectively. The authors believed that some children needed CPAP for respiratory support after extubation, but the PEEP value should not be too high [18]. High PEEP should be avoided in critically ill patients or when intraoperative anastomosis was obviously difficult. In this cohort, 2 patients developed pneumothorax after tracheal intubation and CPAP, and their PEEP values were 4 and 5 cmH_2_O, respectively, which were lower than the former report [18]. In addition, Piyush [21] believed that application of CPAP after extubation after EA/TEF repair would not increase the risk of anastomotic leakage, and our data also supported this view (as shown in Supplementary Table 2). Rational application of CPAP after surgery can help patients to go offline. Therefore, based on the results of this study and previous reports, we still cannot clearly determine the relationship between CPAP and pneumothorax.

There are some limitations in this study. Surgical details, and perioperative management (especially the use of mechanical ventilation) changed over time, and access to surgical details and postoperative complications information was limited. Furthermore, the sample size in a single center and the high proportion of patients who gave up and who were lost to follow-up also limited the conclusions in this study. Thus, the relationship between mechanical ventilation and pneumothorax still needs to be further explored in future multi-center studies with a large sample size.

## Conclusions

The incidence of pneumothorax after EA/TEF repair is about 45 %, and postoperative anastomotic leakage and mechanical ventilation are independent risk factors for the occurrence of pneumothorax. However, the main parameters of mechanical ventilation have no clear correlation with the occurrence of pneumothorax. After timely symptomatic treatment, patients with pneumothorax can have a good prognosis.

## Supplementary Information

**Additional file 1.**

**Additional file 2.**

## Data Availability

The data that support the findings of this study are available from corresponding author but restrictions apply to the availability of these data, which are used under license for the current study, and so are not publicly available. Data are however available from the authors upon reasonable request and with permission of corresponding author.

## Abbreviations

EA/TEF: esophageal atresia/tracheoesophageal fistula
ICU: intensive care unit
PIP: peak inspiratory pressure
PEEP: positive end-expiratory pressure
FiO_2_: fraction of inspiration O_2_
CPAP: continuous positive airway pressure
