# Supplementary material for: Retrospective analysis of pneumothorax after repair of esophageal atresia/tracheoesophageal fistula
Source: BMC Pediatr. 2021 Dec 3;21:543. doi: 10.1186/s12887-021-02948-x (PMC8641193; doi:10.1186/s12887-021-02948-x)
Supplement: Supplementary file 1 — Additional file 1. [file 12887_2021_2948_MOESM1_ESM.docx]

Supplementary Table 1. Clinical comparison between operation approach and postoperative mechanical ventilation

| Variables |  | Operation approach | | *P* |
| --- | --- | --- | --- | --- |
|  |  | Extrapleural | Intrapleural |  |
| Postoperative mechanical ventilation (n, %) | Yes | 60 (46.87) | 68 (53.13) | 0.035* |
|  | No | 38 (63.33) | 22 (36.67) |  |

**The difference was statistically significant*
