# Supplementary material for: Retrospective analysis of pneumothorax after repair of esophageal atresia/tracheoesophageal fistula
Source: BMC Pediatr. 2021 Dec 3;21:543. doi: 10.1186/s12887-021-02948-x (PMC8641193; doi:10.1186/s12887-021-02948-x)
Supplement: Supplementary file 2 — Additional file 2. [file 12887_2021_2948_MOESM2_ESM.docx]

Supplementary Table 2. Clinical comparison between anastomotic leakage and non- anastomotic leakage groups

| Variables | Anastomotic leakage | | *P* |
| --- | --- | --- | --- |
|  | Yes | No |  |
| Postoperative mechanical ventilation | 37 | 69 | 0.136 |
| Continuous positive airway pressure (CPAP) | 9 | 11 | 0.957 |
